# Protecting rare and endangered species under climate change on the Qinghai Plateau, China

**DOI:** 10.1002/ece3.4761

**Published:** 2018-12-10

**Authors:** Renqiang Li

**Affiliations:** ^1^ Key Laboratory of Ecosystem Network Observation and Modeling, Institute of Geographic Sciences and Natural Resources The Chinese Academy of Sciences Beijing China

**Keywords:** climate change, conservation strategies, endangered species, Maxent, Qinghai Plateau, species gain, species richness, species turnover

## Abstract

Climate change‐induced species range shift may pose severe challenges to species conservation. The Qinghai‐Tibet Plateau is the highest and biggest plateau, and also one of the most sensitive areas to global warming in the world, which provides important shelters for a unique assemblage of species. Here, ecological niche‐based model was employed to project the potential distributions of 59 key rare and endangered species under three climate change scenarios (RCP2.6, RCP4.5 and RCP8.5) in Qinghai Province. I assessed the potential impacts of climate change on these key species (habitats, species richness and turnover) and effectiveness of nature reserves (NRs) in protecting these species. The results revealed that that climate change would shrink the geographic ranges of about a third studied species and expand the habitats for two thirds of these species, which would thus alter the conservation value of some local areas and conservation effectiveness of some NRs in Qinghai Province. Some regions require special attention as they are expected to experience significant changes in species turnover, species richness or newly colonized species in the future, including Haidong, Haibei and Haixi junctions, the southwestern Yushu, Qinghai Nuomuhong Provincial NR, Qinghai Qaidam and Haloxylon Forest NR. The Haidong and the eastern part of Haibei, are projected to have high species richness and conservation value in both current and future, but they are currently not protected, and thus require extra protection in the future. The results could provide the first basis on the high latitude region to formulate biodiversity conservation strategies on climate change adaptation.

## 1 INTRODUCTION

Rapid climate change is becoming one of the greatest drivers threatening biodiversity, along with other threats triggered by human‐driven land‐use change (Leach, Zalat, & Gilbert, 2013; Parmesan, 1996). It is projected to become a more significant threat in the coming decades, as many physiological and ecological processes will be affected by global warming and changing precipitation pattern (Deb, Phinn, Butt, & McAlpine, 2017; Hansen et al., 2006). Empirical evidence suggests that climate change has continued to result in the serious degradation or loss of species habitats, and species extinction, especially local species extinction over the past decades (Gong, Guan, Hou, Liu, & Zhou, 2017; Santangeli, Rajasärkkä, & Lehikoinen, 2016; Thomas, Cameron, & Green, 2004). The ancient ecological record of climate change and the model simulation of future species distribution both confirm that climate change is unprecedentedly altering the biodiversity spatial patterns on earth, which has brought serious challenges for biodiversity conservation (Game, Lipsett‐Moore, Saxon, Peterson, & Sheppard, 2011, Gillson, Dawson, Jack, & Mcgeoch, 2013; Wan, Wang, & Yu, 2017). Some new conservation strategies in response to climate change are critically required to be able to adapt and allocate financial resources efficiently (Hannah, Belant, Beevert, Gross, & Lawler, 2010; Parmesan et al., 2013; Wang et al., 2018).

Climate change is driving shifts in species ranges (Parmesan et al., 1999) and the redistributions of species pose a huge challenge for the static boundary of current protected area (PA) networks (Chen, Hill, Ohlemüller, Roy, & Thomas, 2011; D'Amen et al., 2011; Zomer, Xu, & Wang, 2015). In order to adapt to climate change, most species will adopt countermeasures for migration, and move out of the PAs, which will counteract the conservation effectiveness of these PAs (Hannah et al, 2007; Hole et al., 2011). Meanwhile, climate change could bring new species into PAs, which will affect the conservation goal of the existing PAs as well as their management effectiveness (Araújo & Rahbek, 2006, Dawson, Jackson, House, Prentice, & Mace, 2011). Therefore, it is crucial to project the impacts of climate change on species habitat and turnover across time and space, which can greatly help conservation managers with development of adaptation strategies aimed at improving the effectiveness of PA networks and reducing the extinct risk of these key endangered species under the rapid climate change (Araújo, Cabeza, Thuiller, Hannah, & Williams, 2004; Dawson et al., 2011; Li, Xu, Wong, Qiu, Sheng, et al., 2015).

The impacts of climate change on species distributions also referred to as “species distribution models”, have been generally assessed through ecological niche models. These niche‐based models project species distributions by analyzing the relationships between species distributions and a number of environmental variables (Synes & Osborne, 2011; Virkkala & Lehikoinen, 2017). Although these relatively simple models may under‐represent complex natural systems by neglecting competitive interactions, species plasticity, adaptation and time‐lag (Davis, Jenkinson, Lawton, Shorrocks, & Wood, 1998; Pearson & Dawson, 2003), with a good understanding of the modeling techniques, and appropriate model validation and testing, they can be regarded as the primary tools for projecting species habitats and extinction risk, evaluating conservation priorities and assessing reserve designs (Akçakaya, Shm, Mace, Stuart, & Hiltontaylor, 2006; Duckett, Wilson, & Stow, 2013; Gallagher, Hughes, & Leishman, 2013). Therefore, they play a critical role in supporting spatial conservation planning, especially when conservation biologists are often pressed to make recommendations about conserving biodiversity based on limited species distribution data under climate change (Addison et al., 2013; Guisan et al., 2013).

Qinghai Province is situated in the northeast of the Qinghai‐Tibet Plateau. As a traditionally sparsely inhabited region with a variety of different climatic zones and natural habitats, it provides important habitats for the Tibetan antelope (*Pantholops hodgsonii*), snow leopard (*Panthera uncia*), *Procapra przewalskii *and other key rare and endangered animals. It is the highest and biggest Plateau, one of the most sensitive regions to climate change in the world (Li, Powers, Xu, Zheng, & Zhao, 2018). Climate change will lead to higher temperatures and more precipitation in most areas in the year 2061–2080 under three RCPs from Global Circulation Model‐HadGEM2‐ES compared with the current climate condition. (Table 1), which could bring severe challenges for the regional biodiversity conservation (Chen et al., 2013; Duo, 2013). However, it has not been clear how climate change might affect the conservation of key rare and endangered species in Qinghai province. For the first time, this paper aims: (a) to project the potential climate change impacts on the habitats of the key rare and endangered species, species richness, and species turnover in Qinghai province; (b) to assess the efficacy of the existing nature reserves (NRs) for protecting these key species under future climate change; and (c) to comprehensively propose the adaptation strategies of biodiversity conservation in Qinghai province.

**Table 1:** Changes in average temperature and precipitation in the year 2061–2080 under three RCPs from Global Circulation Model‐HadGEM2‐ES, compared with baseline year 1950–2000 in Qinghai Province

Scenario	Temperature (°C)			Precipitation(mm)		
	Min	Mean	Max	Min	Mean	Max
RCP2.6	1.3	2.3	2.9	−6	32	87
RCP4.5	2.4	3.1	4.2	4	44	117
RCP8.5	4.3	5.0	6.3	−18	61	163

## 2 MATERIALS AND METHODS

### 2.1 Study area and species

**Figure 1:**
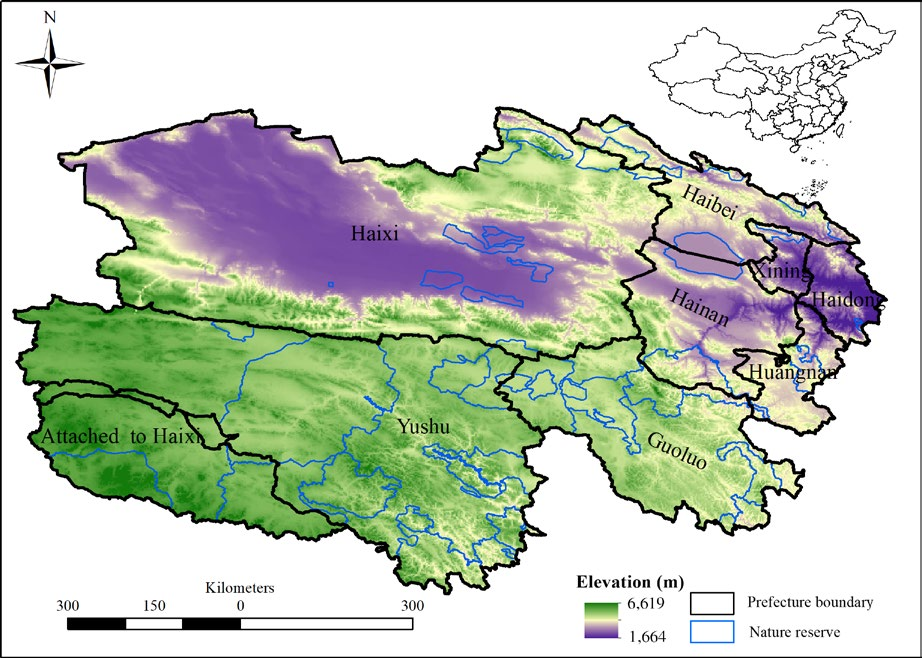
Distributions of nature reserves and Prefectures in Qinghai Province, showing its location in China and the elevation range

Qinghai Province, the “water tower” of China and Asia, is located on the northeast part of Qing‐Tibetan Plateau. It covers an area of over 720,000 square kilometers, one thirteenth of China’s total area. Yangtze River, Yellow River and Lancang River, China's three major rivers all start in Qinghai province (Fang, 2013). Qinghai Province is administratively divided into eight prefecture‐level divisions: two prefecture‐level cities (Xining and Haidong) and six autonomous prefectures (Hainan, Haibei, Huangnan, Yushu, Guoluo, and Haixi).The elevation in this Province ranges from 1,664 to 6,619 m, and its average elevation is over 3,000 m above sea level (Figure 1). Most of the area is situated over 4,000 m above sea level—including the Qilian, Kunlun, Tanggula and other high mountain ranges.

In this study, I integrated species distribution data from two sources to achieve maximum representation of biodiversity and compensate for limitations in data availability on the high latitude region: (a) China key rare and endangered species database collected by The Nature Conservancy’s China biodiversity blueprint project. This database has been successfully used to predict climate change‐induced range shifts of Galliformes in China (Li, Tian, & Li, 2010) and endemic and endangered species in China (Li et al., 2013). It was once employed to identify conservation priority areas in “China national biodiversity conservation strategy and action plan (2011–2030)” (Ministry of Environmental Protection 2010); (b) Chinese Endangered Species Information System (CESIS; Xie, Chen, Zou, Wang, & Wang, 1997). This system collected the latest endangered species information including mammals, birds, reptiles, amphibians, fish species or subspecies in China. Both the theoretical and practical simulations show that when the number of species presence points is >14, the species distribution model can produce a better simulation result of species habitat (Proosdij, Sosef, Wieringa, & Raes, 2016). Therefore, I excluded these species with <15 presence points from the two databases, and obtained species presence data for 59 key rare and endangered animal species, which represents the indicator species of biodiversity conservation in Qinghai Province (see details in Li et al., 2018). Among these species, there were 39 species with more than 100 presence points, and 51 species with over 50 presence points. The minimum number of occurrences was 21.

### 2.2 Predictor variables

A set of 19 bioclimatic variables (Supporting Information Table S2) at 30 s resolution were obtained from the Worldclim database (Hijmans, Cameron, Parra, Jones, & Jarvis, 2005) for current climate (1950–2000) and future climate scenarios for 2070 (average for 2061–2080). The data applied here are the recent IPCC‐CMIP5 climate projections from five Global Circulation Models (GCMs; CCSM4, CNRM‐CM5, HadGEM2‐ES, MIROC5 and MPI‐ESM‐LR) under three representative concentration pathways (RCP 2.6, 4.5 and 8.5). To minimize overfitting of the models, I calculated inter‐correlations among 19 bioclimatic variables, and removed one of the two variables when correlation coefficient > |0.70| was obtained. Consequently, eight bioclimatic variables were used to construct species distribution models(bio2, bio4, bio5, bio8, bio12, bio15, bio17 and bio18; Li et al., 2018; Li, Xu, Wong, Qiu, Sheng, et al., 2015). Slope and aspect were derived from a DEM with a resolution of 90 m, which was obtained from USGS. Global human footprint index at 1 km resolution was collected as human interference data, which integrates disturbance variables such as land‐use change, infrastructure and population density (Sanderson, Jaiteh, Levy, Redford, & Wannebo, 2002). Because reliable future projection of human footprint index is not available, and including static variables in models alongside dynamic variables can improve model performance (Li, Xu, Wong, Qiu, Sheng, et al., 2015), I kept these variables static in our projections.

### 2.3 Species distribution modeling and testing

The maximum entropy approach (Phillips, Anderson, & Schapire, 2006) was employed to project habitat suitability for 59 rare and endangered species on Qinghai Plateau, which has shown to be one of the best performing models in predicting species distributions with presence‐only data (Elith et al., 2006; Hijmans & Graham, 2006), and it has been extensively applied to project species range and vegetation shifts under climate change (Ponce‐Reyes et al., 2012; Rebelo, Tarroso, & Jones, 2010; Wong, Li, Xu, & Long, 2013). The full extent of the study area was used to extract 10,000 pseudo‐absence points to improve model performance (Li, Xu, Wong, Qiu, Sheng, et al., 2015; VanderWal, Shoo, Graham, & Williams, 2009). I built the distribution model for the each species using the selected eight bioclimatic variables, and three environmental variables (slope, aspect, and human footprint index) as predictors. A total of five GCMs were used to produce probability outputs for each scenario. I performed 10 replications and a maximum of 500 iterations for each species, using a cross‐validation procedure where I divided our dataset using 75% of the data for model calibration and retaining 25% of the data for evaluation. I calculated the average predicted probability of occurrence across the five GCMs for each grid as our ensemble forecast (Hole et al., 2009; Li, Xu, Wong, Qiu, Li, et al., 2015; Marmion, Parviainen, Luoto, Heikkinen, & Thuiller, 2009). Subsequently, I applied the Maximum Training Sensitivity Plus Specificity as the threshold to define the presence–absence distribution of species habitats, as this method has been found to be a robust approach (Fajardo, Lessmann, Bonaccorso, Devenish, & Muñoz, 2014; Liu, Berry, Dawson, & Pearson, 2005). The Areas under the Operating Characteristic Curve (AUC), a widely‐used approach, was adopted to evaluate the model performance of our species models. As AUC is not appropriate to evaluate the accuracy of binary predictions, I also used true skill statistic (TSS) as suggested by recent studies (Li & Guo, 2013; Lobo, Jiménez‐Valverde, & Real, 2008) to assess the accuracy of the studied species models. The TSS takes into account both omission and commission errors, and success as a result of random guessing and ranges from −1 to +1, where +1 indicates perfect agreement and values of zero or less indicate a performance no better than random. It is a simple and intuitive measure for the performance of species distribution models when predictions are expressed as presence–absence maps (Allouche, Tsoar, & Kadmon, 2006; Mainali et al., 2016). I used both presence and background data for calculating AUC and TSS. The true absence data was unavailable in our study, so I used the randomly extracted background points within the whole study area for ROC analysis and calculate AUC and TSS (Please see Supporting Information Table S1).

### 2.4 Assessment of climate change impacts in species habitats and assemblages

To estimate the sensitivity to climate change at the species level, I intersected the current and future habitat distribution maps to calculate the potential changes of species habitats. This allowed us to identify areas of the habitat range that are projected to be lost, gain or remain under future climate scenarios. Secondly, two indicators were chosen to evaluate the impact of climate change on species assemblages, including species richness and species turnover. Species richness was generated by calculating the number of species present in each 1‐km^2^ grid cell across the entire study region based on the binary distribution maps produced for the 59 species. Additionally, species turnover was also calculated (Broennimann et al., 2006) from a modification of the “classical” species turnover (beta‐diversity) indicator (Lennon, Koleff, Greenwood, & Gaston, 2001; Whittaker, 1960). This index was measured in geographic space using a defined spatial neighborhood according to Equation (1).

(1) $$\text{Species turnover}=\frac{100\times (\text{species gain}+\text{species loss})}{\left(\text{initial species richness}+\text{species gain}\right)}$$

This index is usually used to measure the intensity of species change in a region with a range from 0 to 100 (Ramirez‐Villegas et al., 2014). It has a lower limit of zero when “species gain” and the “species loss” are both zero (this is generally not possible to happen), and 100 represents a complete species change from one period to another (i.e., the species gain or loss equals to the initial species richness and there is no loss or gain, respectively).

### 2.5 Evaluation of nature reserve effectiveness under climate change

I overlaid the current and future species habitat maps with the boundary of NRs to explore the changes of climatically suitable habitat for each endangered species within NRs under climate change (Araújo et al., 2004; Velásquez‐Tibatá, Salaman, & Graham, 2013). It is crucial for improving conservation effectiveness to evaluate the impacts of climate change on species richness and turnover across time within conservation networks (Hole et al., 2009). The Qinghai NR network includes 45 separate polygon boundaries of conservation subareas (Figure 1). In order to illustrate the conservation effectiveness of different regions and assess the change of species assemblages, I also conducted a separate analysis of the average change in species richness and turnover for each conservation subarea from the current to the future period.

## 3 RESULTS

### 3.1 Accuracy of species distribution models

All models for the 59 rare and endangered species achieved good or excellent performance, with high‐average AUC scores and low omission rates (OR) at the 10% cumulative threshold value, indicating that these models had a high level of accuracy. According to the AUC model assessment criteria: 0.9–1.0 is excellent; 0.8–0.9, good; 0.7–0.8, general; 0.6–0.7, poor; 0.5–0.6, poor (Swets, 1988). All models have high test AUC values (0.878–0.978), which indicated that our models can reasonably capture species‐climate relationships, and thus can be used to project the future habitat suitability of these studied species in Qinghai Province. The high TSS scores of these models also suggested that only a small percentage of test points fell outside the area predicted as “presence”, and our projections have high accuracy.

### 3.2 Climate change impacts on species habitats

**Figure 2:**
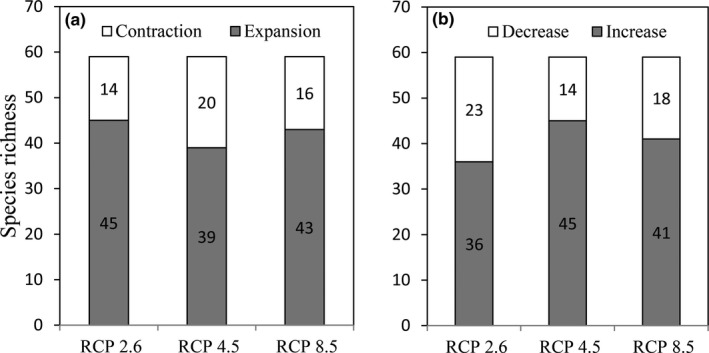
(a) Habitat change (expansion or contraction) of key rare and endangered species in Qinghai Province in the year 2061–2080 under three RCPs (2.6, 4.5 and 8.5); (b) the ability of nature reserves for protecting the key rare and endangered species in Qinghai Province in the year 2061–2080 under three RCPs compared with the current climate conditions

Climate change would have great impacts on the habitats of the 59 rare and endangered species in Qinghai Province. It would lead to the increase of habitats for about 2/3 studied species and the reduction of habitats for about 1/3 species (Supporting Information Table S3). Specifically, under RCP2.6 climate change scenario, 45 species are projected to increase their suitable habitats, and 14 species would lose their current habitats by 2070; Under RCP4.5 scenario, 39 species would expand their suitable habitats, and 20 species would suffer from habitat contraction. Under RCP 8.5 scenario, 43 species are projected to increase their current suitable habitats, and 16 species would have habitat decline in future (Figure 2a). Under the three RCPs, the species with the most habitat loss included *Tetraogallus himalayensis*,* Gervus albirostris*,* Pantholops hodgsonii*,* Tetraogallus tibetanus*, *Equus kiang*, and* Procapra picticaudata.* The species with the most habitat gain included *Neofelis nebulosa*,* Bos mutus*,* Pseudois nayaur*,* Ithaginis cruentus* and* Panthera uncial.*

### 3.3 The impacts of climate change on spatial pattern of species richness

**Figure 3:**
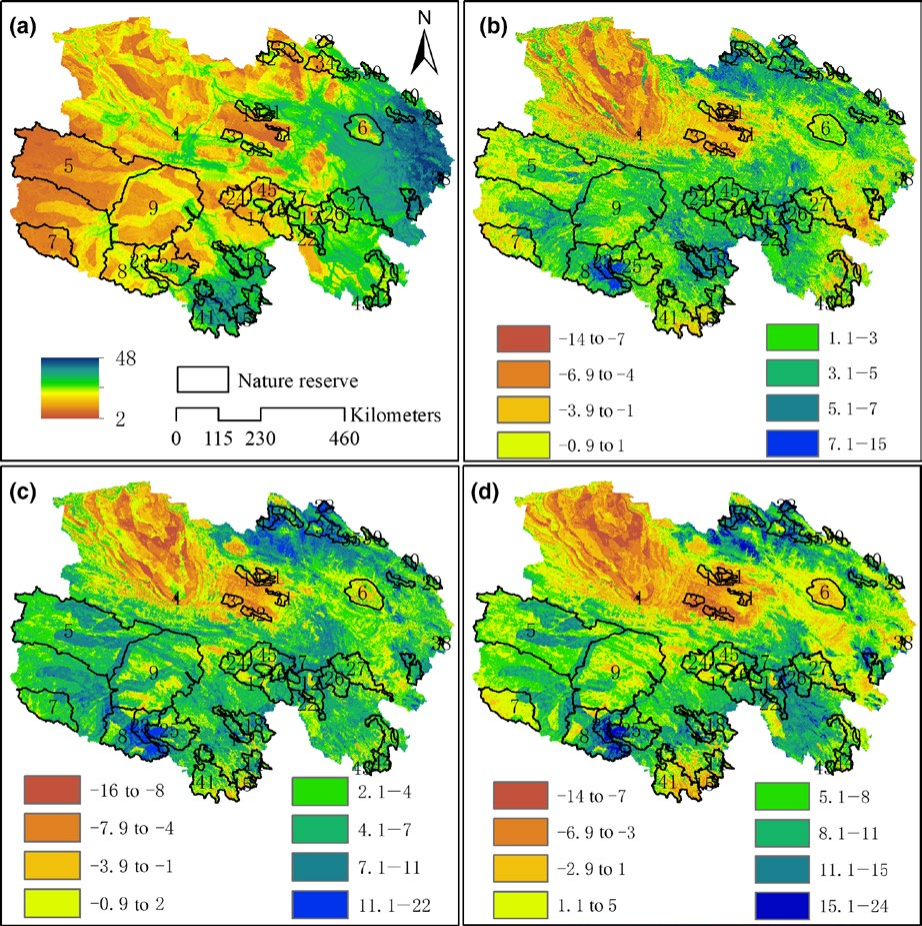
Species richness under current climate conditions (a) and spatial patterns of changes in species richness for key rare and endangered species within and outside of nature reserve of Qinghai Province in the year 2061–2080 under three RCPs compared with baseline year 1960–1990, (b) RCP2.6; (c) RCP4.5; and (d) RCP 8.5

The spatial pattern of current species richness shows a general reduction from low altitude in southeast to northwest high altitude (Figure 3a). The maximum value of species richness with 48 is found to occur in the east, north‐east, and south of Qinghai Province. Under future climate scenarios, species richness is predicted to increase in most areas by 2070, but the spatial pattern was similar to that under the current climate condition and species richness still reduce from the southeast to the west (Figure 3). Species richness of the Qaidam Basin in northwestern Qinghai province decreased greatly, probably due to rapid temperature rise and the little change of precipitation in this area. Species had to move out of this area in order to find climatically suitable habitats and at the same time, the region is a desert area and there are few species survived there. The areas with the obvious increase of species richness are mainly concentrated in Haixi and Haibei Prefectures at the junction of the southwest area of Qilian mountain area and Yushu Prefecture, because the precipitation and temperature is projected to increase comparatively moderately and most species would find climatically suitable habitats in these areas (Figure 3b–d).

**Figure 4:**
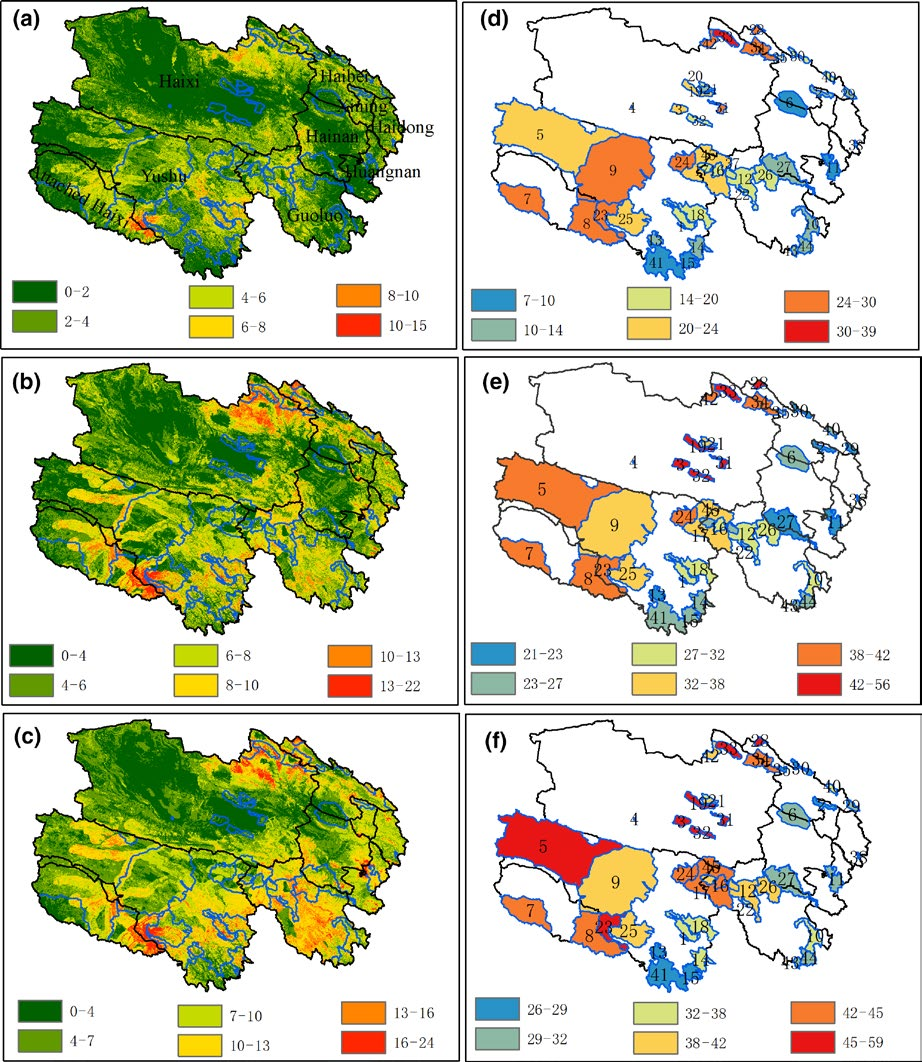
Spatial distribution of the number of species gain in Qinghai Province in the year 2061–2080 under three RCPs: (a) RCP2.6, (b) RCP4.5, (c) RCP8.5; Species turnover for key rare and endangered species in the current nature reserves in the year 2061–2080 under three RCPs compared with baseline year 1950–2000, (d) RCP2.6, (e) RCP4.5, (f) RCP8.5

Species gain would emerge in most parts of Qinghai Province with the intensification of climate change. Under three scenarios, the richness of specie gain could reach 15–24 per km^2^ in some areas, and spatial patterns of these species gain are very similar. The areas with the most new species are mainly located in the border area between the northwest of Haibei Prefecture and Haixi Prefecture, the southwestern region of Yushu, the eastern part of Huangnan Prefecture, and some parts of Guoluo Prefecture (Figure 4a–c).

### 3.4 Conservation effectiveness and species turnover of NR network

Climate change‐induced species range shifts would alter the conservation effectiveness of NR network in protecting these endangered species. The results showed the percentage of habitat in the current NR network for 14–23 species would decrease, while that for 36–45 species would increase under the three climate scenarios. Specifically, under the RCP2.6 scenario, the percentage of habitat in NRs for 23 species would decrease, while that for 36 species would increase. Under RCP4.5 scenario, percentage of 45 species habitats covered by NRs would increase, and it would decrease for 14 species. For RCP 8.5 scenario, it is projected to increase for 41 species and decrease for 18 species.

Future climate change would greatly affect the average species turnover in NRs. Under the three RCPs, NRs with the relatively low species turnover were located in Qinghai Haibei Tibetan Autonomous Prefecture, Qinghai Yushu Tibetan Autonomous Prefecture, and Haixi Mongolian‐Tibetan Autonomous Prefecture. The NRs with the greatest species turnover included Qinghai Nuomuhong Provincial NR, Qinghai Chaidamu Haloxylon Ammodendron Forest National NR, and some conservation subareas of Sanjiangyuan National NR (Tuanjiefeng conservation subarea, Heihe Heyuanyuan conservation subarea, Yueguzonglie conservation subarea, Sanheyuan conservation subarea, Dangqu conservation subarea, and Geladandong conservation subarea; Figures 3d4f).

## 4 DISCUSSION

This study adopted niche‐based models to project potential distribution pattern of the key rare and endangered species in Qinghai Province, and explored the adaptation conservation strategies under climate change. The results suggest that climate change would lead to the expansion of most rare and endangered species habitats, and the shrinking of a few species habitats. This is different from most of the existed research results about the impact of climate change on species habitats in other regions (Warren et al, 2013). There are two possible reasons for this simulation results: (a) Qinghai province is located at a high altitude, and climate change would lead to more climatically suitable habitats in this region; (b) It is assumed in the process of simulation that these animal species could freely migrate to any new climatically suitable areas. However, due to the impact of natural and man‐made barriers on species dispersal and migration, as well as the destruction and restrictions of habitat conditions outside NRs, most species would possibly confront habitat contraction under future climate change. Limited dispersal scenario for species was not considered in this study. Universal dispersal means that species could disperse to any suitable places for population persistence in future, while limited dispersal means that species only can inhabit only places that are modeled to be suitable both in the present and in future (Li, Xu, Wong, Qiu, Sheng, et al., 2015; Thomas et al., 2004). Therefore, universal dispersal scenario was regarded as providing more useful information for implementing human‐assisted adaptation to climate change in future conservation planning.

Under the assumption of species universal dispersal, the spatial distribution pattern of species richness and turnover would experience great changes due to the shifts of species habitats, although the conservation effectiveness of NR network as a whole would not be changed too much under the future climate change. However, climate change may have a great impact on the conservation effectiveness of NRs in some local areas of Qinghai Province. Future conservation strategies should focus on those areas with relatively large changes. In June 2016, China's first national park system pilot was started in the Sanjiangyuan area of the Qinghai‐Tibet Plateau, which would be the world's biggest national park. Our study can provide basic information on climate change impacts for adjusting the existing NRs and developing the first National Park in China, aiming eventually to protect important natural ecosystems and wildlife and ensure sustainable use of natural resource in Qinghai Province.

First, most of the existing NRs are located in the southern part of Qinghai Province to protect plateau alpine meadow and wetland, which fail to fully represent the species richness in the eastern part of Qinghai Province. This simulation results show that under both current and future climate conditions, a great number of rare and endangered species inhabit the Haidong and the eastern part of Haibei Tibetan Autonomous Prefecture in Qinghai province, which have higher species richness and conservation value, and will become one of the areas with high species richness in the future. Judging from the conservation efficiency of unit area, these areas should be the focal points of the biodiversity conservation in Qinghai province under climate change.

Secondly, future conservation strategies should focus on areas with more new species, including Dangqu Conservation Subarea and Guozongmu Conservation Subarea in Sanjiangyuan NR, Dangheyuan Conservation Subarea and Heiheyuan Conservation Subarea in Qilian Mountain NR, as well as the border area between Haibei Tibetan Autonomous Prefecture and Haixi Mongolian Autonomous Prefecture. These areas should implement the climate change adaptation conservation planning, including the establishment of new NRs or dynamic adjustment of NR boundary. Finally, some NRs would face bigger species turnover under climate change, and meanwhile, they would also win more increase in the number of species. The area with higher species turnover means the severer change than other areas. Therefore, biodiversity conservation strategy in the future should increase the investment and enhance management in corridor construction between these NRs and their surrounding areas to improve the connectivity and their ability to adapt to climate change and the migration of species dispersal. These areas include: Qinghai Nuomuhong Provincial NR, Qinghai Chaidamu Haloxylon ammodendron Forest NR, and Tuanjiefeng Conservation Subarea in Sanjiangyuan National NR.

Although our models indicate good predictive ability, there are several sources of uncertainties in adopting current species–climate relationships to project future climate conditions of species niches. First, there is a lack of sufficient information on biodiversity in Qinghai province. In particular, the habitat characteristics and threat factors of many endangered species are not completely clear. The ongoing investigation of animal and plant resources in Qinghai Province will help to understand the current status of key protected species, including population size, habitat distribution and threat level, which could further improve the accuracy of future assessment. Moreover, it is difficult for the current equilibrium simulation method to take into account any biotic interactions, such as competition with other species or other individuals, predation, and changes in food availability, which may lead to some uncertainty. Furthermore, the species distribution models cannot account for acclimation and adaptation of different species to future climate change. In reality, evolution and adaptation of species could be rapid and potentially help them counter stressful conditions or realize ecological opportunities arising from climate change. Thus, a future direction for improving predictive accuracy should incorporate evolutionary considerations and interspecific relationship of species, into predictive modeling or exploring the climate adaptive capacity of species to climate change. In spite of this, this study employed widely‐used method to conduct the first preliminary assessment of climate change impacts on rare and endangered species in Qinghai Province, which can provide a key reference for adapting biodiversity conservation strategies to climate change on the Qinghai Plateau.

## CONFLICT OF INTEREST

None declared.

## AUTHOR CONTRIBUTIONS

R.L. conceived and completed this study.

## DATA ACCESSIBILITY

Data will be available at the external repository Dryad.
