# Supplementary material for: Protecting rare and endangered species under climate change on the Qinghai Plateau, China
Source: Ecol Evol. 2018 Dec 10;9(1):427–36. doi: 10.1002/ece3.4761 (PMC6342101; doi:10.1002/ece3.4761)
Supplement: Supplementary file 1 [file ECE3-9-427-s001.docx]

**Table S1**. Summary of species data source for each species including scientific name, conservation status, and the number of presence points in this study.

| No. | Presence points | Scientific name | Level | AUC | TSS |
| --- | --- | --- | --- | --- | --- |
| 1 | 43 | *Tetraogallus himalayensis* | LC | 0.8781 | 0.6694 |
| 2 | 195 | *Gervus albirostris* | LC | 0.8802 | 0.7207 |
| 3 | 34 | *Aquila heliaca* | LC | 0.8784 | 0.75 |
| 4 | 26 | *Pelecanus onocrotalus* | VU | 0.8805 | 0.6691 |
| 5 | 31 | *Bonasa sewerzowi* | LC | 0.8906 | 0.7139 |
| 6 | 425 | *Panthera pardus* | EN | 0.8808 | 0.7313 |
| 7 | 133 | *Pantholops hodgsonii* | VU | 0.8917 | 0.6111 |
| 8 | 55 | *Tetraogallus tibetanus* | LC | 0.8827 | 0.6787 |
| 9 | 79 | *Equus kiang* | LC | 0.8856 | 0.7692 |
| 10 | 123 | *Procapra picticaudata* | VU | 0.8929 | 0.7263 |
| 11 | 105 | *Aquila nipalensis* | LC | 0.9061 | 0.7616 |
| 12 | 207 | *Cuon alpinus* | VU | 0.897 | 0.6842 |
| 13 | 718 | *Vulpes vulpes* | VU | 0.8816 | 0.7742 |
| 14 | 122 | *Otis tarda* | LC | 0.8847 | 0.6923 |
| 15 | 179 | *Buteo hemilasius* | LC | 0.8964 | 0.7255 |
| 16 | 185 | *Andrias davidianus* | LC | 0.8867 | 0.6768 |
| 17 | 128 | *Cygnus cygnus* | NE | 0.8911 | 0.7107 |
| 18 | 94 | *Gazella subgutturosa* | LC | 0.921 | 0.6468 |
| 19 | 77 | *Pandion haliaetus* | CR | 0.9557 | 0.7222 |
| 20 | 96 | *Gyps himalayensis* | VU | 0.879 | 0.7309 |
| 21 | 344 | *Milvus lineatus* | LC | 0.8621 | 0.6524 |
| 22 | 277 | *Ciconia nigra* | CR | 0.8846 | 0.8131 |
| 23 | 111 | *Grus nigricollis* | VU | 0.8804 | 0.6746 |
| 24 | 225 | *Ursus thibetanus* | LC | 0.8951 | 0.7647 |
| 25 | 503 | *Chrysolophus pictus* | LC | 0.887 | 0.7502 |
| 26 | 248 | *Falco tinnunculus* | LC | 0.8826 | 0.7251 |
| 27 | 52 | *Gypaetus barbatus* | CR | 0.8895 | 0.7836 |
| 28 | 573 | *Mustela sibirica* | NT | 0.8907 | 0.801 |
| 29 | 110 | *Grus grus* | VU | 0.8915 | 0.7998 |
| 30 | 72 | *Crossoptilon auritum* | NT | 0.8946 | 0.7037 |
| 31 | 506 | *Canis lupus* | LC | 0.8965 | 0.7671 |
| 32 | 48 | *Falco cherrug* | NT | 0.9012 | 0.7937 |
| 33 | 504 | *Capricornis rubidus* | VU | 0.9033 | 0.7774 |
| 34 | 47 | *Lophophorus lhuysii* | LC | 0.9095 | 0.7118 |
| 35 | 246 | *Cervus elaphus* | EN | 0.9119 | 0.7894 |
| 36 | 116 | *Moschus chrysogaster* | LC | 0.9223 | 0.8137 |
| 37 | 653 | *Macaca mulatta* | LC | 0.9227 | 0.7792 |
| 38 | 130 | *Ovis ammon* | VC | 0.9233 | 0.8366 |
| 39 | 21 | *Procapra przewalskii* | LC | 0.9262 | 0.8148 |
| 40 | 297 | *Accipiter nisus* | LC | 0.9302 | 0.7917 |
| 41 | 269 | *Lynx lynx* | LC | 0.9333 | 0.7926 |
| 42 | 140 | *Martes foina* | LC | 0.9336 | 0.793 |
| 43 | 318 | *Cervus unicolor* | LC | 0.9341 | 0.9048 |
| 44 | 552 | *Lutra lutra* | LC | 0.9411 | 0.8105 |
| 45 | 105 | *Antropoides virgo* | VU | 0.942 | 0.8613 |
| 46 | 225 | *Aegypius monachus* | NT | 0.9445 | 0.8887 |
| 47 | 144 | *Otocolobus manul* | VU | 0.9469 | 0.8559 |
| 48 | 95 | *Marmota himalayana* | LC | 0.9498 | 0.8762 |
| 49 | 171 | *Mustela altaica* | LC | 0.9581 | 0.8786 |
| 50 | 319 | *Ailurus fulgens* | EN | 0.959 | 0.8696 |
| 51 | 161 | *Panthera uncia* | NT | 0.9619 | 0.8847 |
| 52 | 85 | *Ithaginis cruentus* | LC | 0.9666 | 0.8889 |
| 53 | 183 | *Pseudois nayaur* | LC | 0.971 | 0.9053 |
| 54 | 91 | *Falco subbuteo* | VU | 0.9721 | 0.9334 |
| 55 | 104 | *Bos mutus* | LC | 0.9728 | 0.8148 |
| 56 | 41 | *Cygnus olor* | LC | 0.9751 | 0.5151 |
| 57 | 77 | *Falco peregrinus* | VU | 0.9764 | 0.9625 |
| 58 | 75 | *Haliaeetus leucoryphus* | VU | 0.9781 | 0.8974 |
| 59 | 292 | *Neofelis nebulosa* | VU | 0.9784 | 0.9054 |

**Table S2.** The description of 19 bioclimatic variables from WorldClim Version2

| Bioclimatic variables | Description |
| --- | --- |
| Bio1 | Annual Mean Temperature |
| Bio2 | Mean Diurnal Range (Mean of monthly (max temp - min temp)) |
| Bio3 | Isothermality (BIO2/BIO7) (* 100) |
| Bio4 | Temperature Seasonality (standard deviation *100) |
| Bio5 | Max Temperature of Warmest Month |
| Bio6 | Min Temperature of Coldest Month |
| Bio7 | Temperature Annual Range (BIO5-BIO6) |
| Bio8 | Mean Temperature of Wettest Quarter |
| Bio9 | Mean Temperature of Driest Quarter |
| Bio10 | Mean Temperature of Warmest Quarter |
| Bio11 | Mean Temperature of Coldest Quarter |
| Bio12 | Annual Precipitation |
| Bio13 | Precipitation of Wettest Month |
| Bio14 | Precipitation of Driest Month |
| Bio15 | Precipitation Seasonality (Coefficient of Variation) |
| Bio16 | Precipitation of Wettest Quarter |
| Bio17 | Precipitation of Driest Quarter |
| Bio18 | Precipitation of Warmest Quarter |
| Bio19 | Precipitation of Coldest Quarter |

**Table S3.** Habitat area, rate of change and protection ratio of habitats for each major endangered species in Qinghai Province under climate change scenarios

| No. | Scientific name | Current habitat area  (km2) | Habitat area in 2080 (km2) | Change rate of habitat area（%） | Protection ratio of habitat in current condition (%) | Protection ratio of habitat in 2080 (%) |
| --- | --- | --- | --- | --- | --- | --- |
| 1 | *Tetraogallus himalayensis* | 103078.5 | 40446.8 | -60.8 | 10.3 | 12.5 |
| 2 | *Gervus albirostris* | 57536.1 | 88908.47 | 54.5 | 27.7 | 32.6 |
| 3 | *Aquila heliaca* | 41867.8 | 46099.7 | 10.1 | 21.3 | 22.2 |
| 4 | *Pelecanus onocrotalus* | 39693.3 | 18940.7 | -52.3 | 14.3 | 14 |
| 5 | *Bonasa sewerzowi* | 70714.1 | 94417.3 | 33.5 | 25.7 | 24.2 |
| 6 | *Pantholops hodgsonii* | 47271.7 | 23235.42 | 50.9 | 38.7 | 37.3 |
| 7 | *Tetraogallus tibetanus* | 91661.2 | 144017.2 | 57.1 | 36.8 | 41.2 |
| 8 | *Equus kiang* | 113401.5 | 74544.7 | -34.3 | 39.2 | 40.4 |
| 9 | *Procapra picticaudata* | 15377.3 | 29424.2 | 91.3 | 54.5 | 52.9 |
| 10 | *Aquila nipalensis* | 90639.6 | 86651.3 | -4.4 | 15 | 16.3 |
| 11 | *Cuon alpinus* | 97883.5 | 125472.1 | 28.2 | 23.3 | 25.9 |
| 12 | *Vulpes vulpes* | 71178.6 | 173143.9 | 143.3 | 19 | 31.1 |
| 13 | *Otis tarda* | 1879.7 | 1658.9 | -11.8 | 8.4 | 9.5 |
| 14 | *Buteo hemilasius* | 145011.4 | 243591.8 | 68 | 26.5 | 31.7 |
| 15 | *Andrias davidianus* | 4699.3 | 27048.5 | 475.6 | 2.3 | 10.2 |
| 16 | *Cygnus cygnus* | 41222 | 55120.6 | 33.7 | 11.6 | 12.8 |
| 17 | *Gazella subgutturosa* | 23749.5 | 22834.6 | -3.9 | 2 | 0.7 |
| 18 | *Pandion haliaetus* | 16225.2 | 57847.5 | 256.5 | 31.3 | 42.3 |
| 19 | *Gyps himalayensis* | 86001.7 | 68184.6 | -20.7 | 20.9 | 22 |
| 20 | *Milvus lineatus* | 50886.8 | 67946.6 | 33.5 | 17.3 | 23.1 |
| 21 | *Ciconia nigra* | 5270.5 | 8293.9 | 57.4 | 12.3 | 13.2 |
| 22 | *Grus nigricollis* | 77674.8 | 125069 | 61 | 24.8 | 24.3 |
| 23 | *Ursus thibetanus* | 18051.2 | 39244.8 | 117.4 | 43.8 | 30.1 |
| 24 | *Chrysolophus pictus* | 3076.2 | 17372.4 | 464.7 | 2 | 3.4 |
| 25 | *Falco tinnunculus* | 58967.3 | 108906.1 | 84.7 | 8.7 | 11.4 |
| 26 | *Gypaetus barbatus* | 65766.5 | 48919.1 | -25.6 | 27.8 | 23 |
| 27 | *Mustela sibirica* | 30510 | 64454 | 111.3 | 15.4 | 24 |
| 28 | *Grus grus* | 33484.2 | 61096.5 | 82.5 | 17.3 | 21.2 |
| 29 | *Panthera pardus* | 229.4 | 11687.3 | 4993.8 | 19.3 | 11.8 |
| 30 | *Crossoptilon auritum* | 135076.6 | 144967.1 | 7.3 | 21.9 | 18.5 |
| 31 | *Canis lupus* | 46795.6 | 80277.9 | 71.6 | 21.9 | 30.3 |
| 32 | *Falco cherrug* | 122859.5 | 192829.6 | 57 | 21.6 | 30.2 |
| 33 | *Capricornis rubidus* | 3493.5 | 14948.6 | 327.9 | 72.5 | 42.6 |
| 34 | *Lophophorus lhuysii* | 1535.3 | 5983.4 | 289.7 | 17.7 | 16.7 |
| 35 | *Cervus elaphus* | 129456.7 | 174277.9 | 34.6 | 36.1 | 35.3 |
| 36 | *Moschus chrysogaster* | 81969.9 | 111141 | 35.6 | 20.7 | 20 |
| 37 | *Macaca mulatta* | 19139.9 | 55272.6 | 188.8 | 43.8 | 31.4 |
| 38 | *Ovis ammon* | 49944.5 | 40056.8 | -19.8 | 23.8 | 29.4 |
| 39 | *Procapra przewalskii* | 1124.5 | 847.9 | -24.6 | 3.2 | 2.6 |
| 40 | *Accipiter nisus* | 23674 | 71748.9 | 203.1 | 30.3 | 28.9 |
| 41 | *Lynx lynx* | 125509 | 186519.5 | 48.6 | 26.5 | 29.7 |
| 42 | *Martes foina* | 143088.1 | 172725.7 | 20.7 | 22 | 22.6 |
| 43 | *Cervus unicolor* | 8819.8 | 20942.4 | 137.4 | 47 | 35.2 |
| 44 | *Lutra lutra* | 29563 | 57840.9 | 95.7 | 25.5 | 26.4 |
| 45 | *Antropoides virgo* | 88270.6 | 29269.3 | -66.8 | 5.3 | 9.4 |
| 46 | *Aegypius monachus* | 79532.9 | 67318.7 | -15.4 | 18.5 | 29.2 |
| 47 | *Otocolobus manul* | 127090.5 | 156588.3 | 23.2 | 20.6 | 23.1 |
| 48 | *Marmota himalayana* | 58554.7 | 65754.2 | 12.3 | 16.7 | 15.9 |
| 49 | *Mustela altaica* | 61745.1 | 114240.9 | 85 | 17.7 | 28.1 |
| 50 | *Ailurus fulgens* | 55.7 | 2234.9 | 3911.9 | 100 | 78 |
| 51 | *Panthera uncia* | 34302.7 | 29529.4 | 14.5 | 25.6 | 24.7 |
| 52 | *Ithaginis cruentus* | 24123.4 | 40589.3 | 68.3 | 31.7 | 28.9 |
| 53 | *Pseudois nayaur* | 140544.4 | 184668.9 | 31.4 | 41.7 | 40.7 |
| 54 | *Falco subbuteo* | 96.3 | 557.1 | 478.4 | 46.1 | 23.9 |
| 55 | *Bos mutus* | 111626.4 | 113494 | 1.7 | 29.6 | 30.4 |
| 56 | *Cygnus olor* | 31296.5 | 85605.1 | 173.5 | 32 | 28 |
| 57 | *Falco peregrinus* | 15398 | 20864 | 35.5 | 1.4 | 1.4 |
| 58 | *Haliaeetus leucoryphus* | 54796.7 | 39138.1 | -28.6 | 10.5 | 15.7 |
| 59 | *Neofelis nebulosa* | 3398.2 | 11267.2 | 231.6 | 70.4 | 58.9 |
